# Understanding the processes involved in sepsis recognition across clinical contexts to guide individualised management: A phenomenographic study

**DOI:** 10.1371/journal.pone.0360913

**Published:** 2026-10-27

**Authors:** Marika Nemeckova, Tony Conner, Alison Steven

**Affiliations:** 1 Department of Nursing, Midwifery and Health, Faculty of Health and Life Sciences, Northumbria University, Newcastle upon Tyne, United Kingdom; 2 Department of Health Sciences, School of Medical and Pharmaceutical Sciences, University of Genova, Genova, Italy; University of Oulu, FINLAND

## Abstract

**Background:**

Sepsis is a highly heterogeneous condition, yet it is commonly recognised by application of standardised criteria, irrespective of clinical context. Understanding the complexity of sepsis recognition in the heterogeneous populations of patients with sepsis is critical to better target our resources to the needs of patients. Our aim was to examine nurses’ clinical experiences to understand the processes involved in sepsis recognition across clinical contexts.

**Methods:**

Semi-structured interviews were conducted with 26 nurses with various levels of experience in caring for patients with sepsis from multiple hospital settings. The interviews were phenomenographically analysed using the Arkelind method. The Cynefin framework was used to inform the organisation of findings according to contextual complexity.

**Findings:**

We identified four categories of description that represent different ways of experiencing sepsis recognition: recognise sepsis by following protocol-based care, recognise sepsis by following disease-specific care, recognise sepsis according to emergent care, and recognise sepsis according to emergency care. The categories emerged from various clinical contexts, ranging from the simple to the highly complex. This variability directed clinical management by helping nurses decide whether guidelines apply to the individual patient and how they should be integrated into clinical decision-making.

**Conclusions:**

Despite its apparent simplicity, sepsis recognition is complex and context-dependent. Rather than relying on an identical strategy for all patients, effective clinical decisions require personalisation based on the characteristics of the clinical contexts. Our findings show that considering the availability of data, predictability and cause-and-effect relationships can inform the selection of an appropriate approach to sepsis recognition. This approach demands adaptability; however, unless we adopt an approach that is appropriate to the level of clinical complexity, significant improvements in sepsis recognition may not be seen. Prospective research is required to validate our findings.

## Introduction

Sepsis is a substantial global health problem associated with high incidence and unacceptably high mortality and morbidity [1]. Early identification of sepsis is crucial for improving outcomes [2], to ensure that treatment can be started early to prevent deterioration [3]. However, recognising sepsis in practice remains challenging [4].

There is no gold-standard definition of sepsis, nor are there universally accepted diagnostic criteria in either clinical practice or clinical research [5]. The latest international definition, Sepsis-3, developed by the European Society of Intensive Care Medicine and Society of Critical Care Medicine task force in 2016, defines sepsis as “life-threatening organ dysfunction caused by a dysregulated host response to infection” [5, p.6]. This definition has been operationalised through suspected infection alongside organ dysfunction, assessed using the Sequential Organ Failure Assessment (SOFA) score. Although the quick Sequential Organ Failure Assessment (qSOFA) was introduced as a bedside tool [5], it was intended to predict mortality risk in patients with suspected infection rather than to support the early identification of sepsis [2]. Consequently, there remains a lack of consensus about what constitutes sepsis recognition in practice [4].

International sepsis guidelines have recommended the use of sepsis screening tools designed to promote early identification of sepsis [2], irrespective of clinical context [6]. While such an approach can yield positive outcomes, in many cases it is ineffective [7,8], or can contribute to poor outcomes of septic patients [9], due to delayed recognition and management of sepsis [3].

A major challenge to protocolising sepsis recognition is that existing screening tools treat sepsis as a homogeneous syndrome [10]. Sepsis is a dynamic condition, and the diagnostic criteria of these tools may not all be present at one point in time [4]. Sepsis exhibits a very broad spectrum of disease manifestations, with no single identified diagnostic element present in all patients [4]. The sepsis criteria are often highly specific and may not apply to the broader range of clinical settings in which sepsis occurs [6]. According to Filbin et al. [11], a third of patients presenting to an emergency department with septic shock had vague symptoms not specific to infection, which are largely neglected in most diagnostic tools.

In addition, efforts to identify patients with sepsis often focus on the use of blood cultures to detect bacteraemia and subsequent sensitivity testing if cultures are positive [4]. Even when cultures are positive, several days may be needed before results are available. This creates uncertainty regarding how best to approach sepsis recognition across different clinical contexts and at different points of clinical progression.

International sepsis guidelines promote the personalisation of clinical decisions [2], but there seems to be a lack of understanding about the way in which personalisation is achieved in sepsis recognition [6]. Current studies focus mainly on the evaluation of existing protocols [7,8], but data on how they should be integrated into clinical decision when exposed to heterogeneous sepsis presentations are lacking.

The challenge is that the personalisation of clinical decisions is complex and demands bedside expertise [6]. Nurses are healthcare professionals directly involved in close observations of the patient’s condition. They observe patients in various clinical situations, particularly in the complex contexts in which a clear disease pattern has not yet developed [12]. Therefore, the aim of the study was to examine nurses’ clinical experiences to understand the processes involved in sepsis recognition across clinical contexts.

An understanding of the processes involved in sepsis recognition at the bedside could illustrate how sepsis screening tools are used in practice. This could verify whether the current standardised approach to sepsis recognition is appropriate or measures anything of significance or value. Such an understanding may allow future sepsis recognition practice to be grounded in a framework of a relevant and personalised approach to sepsis recognition, which is currently lacking [13].

## Methods

This study used a phenomenographic design, chosen to capture variation in how nurses recognise sepsis across clinical contexts [14]. Phenomenography is based on the idea that variation in how a phenomenon is experienced reflects differences in the meanings people ascribe to it within a given context. It enables the identification of categories that reflect varying levels of contextual understanding and sophistication within a group [14].

### Participants and recruitment

Participants were nurses recruited from a variety of clinical departments across two hospitals in a region of the UK. Recruitment took place between 28 January 2021 and 12 August 2021. The recruitment process began with approaching members of the hospital staff who identified all eligible staff members. This was a voluntary opt in study, and participants willing to participate could opt in. An appropriately trained staff member, who is employed by the hospital Trust, circulated information about the study and consent forms to these lists and asked that, if they were willing to participate, they contact the researcher to arrange suitable times for participation in the individual interviews. In addition, posters were placed in clinical areas, publicising the project to nursing staff.

Eligible participants were registered nurses who had experience of caring for patients with sepsis. Purposeful maximal variation sampling was used to investigate the variation in the contexts in which the phenomenon was experienced [15]. The sample size was determined by data saturation, meaning sampling stopped when no additional previously undescribed experiences emerged for several interviews [15].

### Informed consent and ethics

Participants provided informed consent prior to participation. Written informed consent was obtained using an electronic consent process in line with Health Research Authority guidance. Participants were provided with a plain-language information statement before the interview, and consent was obtained and documented by the researcher conducting the interviews. Consent was also verbally reconfirmed at the start of each interview. Copies of the signed consent forms were provided to participants and securely retained by the research team.

Ethics approvals for this research were received from Northumbria University and the Health Research Authority (280396). The present article adheres to the EQUATOR guidelines for reporting research using the Standards for Reporting Qualitative Research (SRQR) checklist (see S1 Checklist) [16].

### Data analysis

Data were analysed using the Åkerlind method [15]. NVivo 12 software was used for data management. The analysis followed a holistic transcript approach because participants’ responses were likely to be contextually situated or contextually confirmed. As shown in Table 1, the analysis involved familiarisation with the data, grouping transcripts according to emerging themes, developing dimensions of variation, organising these dimensions under themes, establishing relationships among the dimensions of variation by identifying similarities and differences, incorporating remaining transcripts, and developing categories of description, which led to the construction of an outcome space. In this study, dimensions of variation were the distinct aspects of sepsis recognition that were present in some transcripts but not in others [15]. Data collection and analysis occurred concurrently, with iterative adjustments to the interview guide and preliminary themes until data saturation was reached [15].

**Table 1:** Data analysis process.

Stage	Description
1	Transcribing interviews; familiarising with data
2	Grouping transcripts by theme
3	Developing dimensions of variation
4	Organising dimensions of variation under themes
5	Establishing relationships among the dimensions of variation
6	Adding remaining transcripts
7	Developing categories of description
8	Developing an outcome space

During the familiarisation process, the interviews were read several times holistically to gain a sense of the whole, as the Åkerlind approach requires the entire interview to be considered [15]. This holistic familiarity of each transcript is the key requirement for the development of accurate themes and dimensions of variation.

The grouping of transcripts was systematically organised according to the theme or context for the described ways of working which emerged during the data familiarisation stage 1. Four different themes were identified. Each theme or context is representative of how participants described their ways of working. A common description was formulated for each theme: (A) protocol-based care; (B) disease-specific care; (C) emergent care and (D) emergency care.

Some transcripts could be interpreted in relation to more than one theme because participants’ accounts often contained more than one way of experiencing sepsis recognition; NVivo 12 allowed each transcript to be located in multiple groups rather than being forced into a single category, thereby preserving the contextual complexity of participants’ accounts during analysis. Åkerlind method recognises the whole transcript as a ‘unit’ of data, at least in the early stages [15]. Consistent with this approach, the whole transcript was retained as the contextual unit during the initial stages of analysis rather than individual excerpts or utterances being extracted into a decontextualised pool of meanings. This holistic approach preserved the contextual situatedness of participants’ accounts.

To move from dimensions of variation to final categories, dimensions of variation were developed through comparison of statements across several transcripts, rather than being derived from single statements [15]. Categories of description were formed by combining related dimensions of variation that reflected a similar degree of complexity. Finally, the outcome space was constructed to represent the relationships between the categories, organising all categories inclusively in relation to one another and generating a comprehensive picture of the different ways of understanding the phenomenon.

The raw phenomenographic content of each category was as follows. Protocol-based care was defined by an emphasis on a protocolised approach to disease, where the human body is viewed as an object against which existing pre-agreed criteria (normal and abnormal observation ranges as indicated in the protocol bundle) are compared. Disease-specific care was established by the primary focus of attention on a reductionist approach to disease, in which a reduction in symptoms and stability of the patient’s condition are seen as key to the early recognition of sepsis. The human body is viewed as the sum of distinct parts, each of which can develop an isolated disease. A disease is then considered to be a result of a malfunction of one of these parts, with a well-defined label that demands professional judgement. Efforts are concentrated on breaking down a patient’s signs and symptoms into individual components and defining, with great accuracy and precision, the intervention required for each malfunction. Emergent care captured a shift in focus from the individual disease to the individual patient, where a patient is considered to have a disease that might manifest in a unique and unpredictable manner. Furthermore, the focus of attention shifted from a highly-protocolised to an emergent approach, where the recognition of nonlinear and frequently unseen connections between variables is highlighted as the key to early recognition of sepsis. Early recognition of sepsis is perceived as more complex because it entails unique and adaptive responses to novel stimuli from an external environment. Emergency care was characterised by a shift to an emergency approach where life-saving measures take precedence over other clinical requirements. Thus, the aim is to stabilise the patient and reduce immediate clinical uncertainty. Each theme is elaborated further, with supporting participant quotations, in the Findings section.

As the data familiarity and analysis progressed, it became clear that this series of qualitatively different ways of experiencing sepsis recognition, emerged from phenomenographic analysis, is highly related to the knowledge management system suggested by Snowden [17]. The Cynefin framework comprises the following domains: simple, complicated, complex and chaotic. Every domain makes use of reasoning pathways to generate knowledge and solve problems [17]. To exemplify this, the reasoning pathway in the complicated domain of the Cynefin framework is analytically based on hypothetical-deductive logic and makes use of explicit knowledge such as facts, rules and procedures [18]. On the other hand, the reasoning pathway in the complex domain is more tacit knowledge-based, and a form of knowledge which is often difficult to articulate. The reasoning pathway is driven by personal perceptions, intuition and beliefs, which are often the result of a mixture of formal training and experience. Many of these characteristics of the framework resonate with the complexity of this study. This characteristic allowed for organising knowledge explored in this study based on the reasoning pathways used by nurses, while still taking into account knowledge creation and its application in practice.

An additional characteristic that resonates with the nature of the findings is that reasoning pathways explored in this phenomenographic study often resided only temporarily in one domain [17]. In some circumstances, nurses operated predominantly in one domain when solving problems, as not every situation required additional reasoning strategies. However, as time continued to pass in assessment without results, other reasoning pathways were often drawn upon to increase the likelihood of a successful outcome. Nurses who operated simultaneously across domains were thinking comprehensively about the problem and used a combination of these four reasoning pathways; in general, the more complex the situation, the greater the likelihood that multiple reasoning strategies were required to resolve it. Reasoning therefore frequently spanned more than one domain within the same account, rather than being confined to a single, discrete domain. Cynefin provided a reference language to dynamically contextualise situations located in different contexts that required distinguished responses and characterised by different patterns of cause-effect relationship [18].

At the initial stage of data analysis, one researcher (MN) independently analysed ten interview transcripts, identifying preliminary ways of experiencing the phenomenon and an initial set of dimensions of variation. These interpretations were then reviewed by one additional member of the research team (TC), who provided detailed feedback. Subsequently, three team members (MN, AS and TC) took part in a consensus meeting to compare interpretations and resolve discrepancies. Disagreements, primarily relating to the boundaries and representation of variations within the data, were addressed through iterative discussion until agreement was reached. MN then analysed the remaining transcripts, with ongoing refinement of the dimensions of variation and emerging categories of description, which were reviewed and agreed by the research team.

### Data collection

In line with phenomenography, data were collected through semi-structured interviews. The first author conducted all interviews herself to ensure consistency in questioning, prompting, and comparison of responses across participants. No repeat interviews were conducted. With participant permission, all interviews were audio-recorded. Interviews were transcribed verbatim. All interviews took place between March and June 2021. Interviews lasted between 25 and 63 minutes. Although interview length varied, all interviews generated sufficiently rich data to support phenomenographic analysis.

Data were collected using an interview guide that provided a general framework for the discussion, while permitting participant exploration of related topics. The interview guide evolved iteratively based on preliminary analyses conducted concurrently with the data collection (see S2 File).

### Rigour and reflexivity

This qualitative study maintained rigour by providing detailed descriptions of all research steps. The use of methods and analysis was carefully recorded. The first author’s background as a nurse supported data interpretation but required careful reflexivity, as the interviewer’s prior experience with sepsis recognition may have influenced data collection and interpretation. To balance these assumptions, interpretations were regularly discussed within the multidisciplinary research team, whose diverse nursing backgrounds enabled collaborative comparison of interpretations and the revisiting of transcripts where differing perspectives arose. Reflexivity discussions in the author group addressed personal, interpersonal, methodological, and contextual matters. Initial analytic development involved ten transcripts analysed by MN, followed by review and team consensus. The remaining transcripts were analysed iteratively, with the final two interviews examined against the established dimensions and categories to confirm that no additional ways of experiencing sepsis recognition emerged.

Participant checking was not conducted because phenomenography focuses on describing the collective and qualitatively different ways people experience a phenomenon rather than verifying an individual’s accuracy [15]. As an alternative, the researchers participated in continuous discussion and routinely revisited transcripts to ensure that the interpretations were supported with the data transcripts. Credibility was strengthened by outlining and demonstrating the connections between the themes and the data through the use of quotations [15].

## Findings

### Sample description

The sample consisted of twenty-six nurses recruited from nine settings across two large hospitals in the North East of England. The sample reflected variation in experience, specialism, and length of time working in the hospital, as shown in Table 2. Data saturation was achieved by the 24th interview, and two further participants were then interviewed to confirm thematic redundancy.

**Table 2:** Participant characteristics.

Participant	Age	Gender	Years of professional experience	Nursing specialism	Nursing qualification
**P 01**	60+	Female	31+	Research	Degree
**P 02**	40-49	Female	6-10	Ward-based care	Degree
**P 03**	40-49	Female	21-30	Research	Degree
**P 04**	20-29	Female	0-5	Research	Degree
**P 05**	20-29	Female	0-5	Critical care	Degree
**P 06**	20-29	Female	0-5	Ward-based care	Degree
**P 07**	20-29	Female	0-5	Critical care	Degree
**P 08**	20-29	Female	0-5	Ward-based care	MSc
**P 09**	50-59	Female	21-30	Critical care	Degree
**P 10**	30-39	Female	11-20	Critical care	Degree
**P 11**	20-29	Female	6-10	Ward-based care	Postgraduate certificate
**P 12**	40-49	Female	21-30	Ward-based care	Postgraduate certificate
**P 13**	30-39	Female	6-10	Critical care	Degree
**P 14**	20-29	Female	6-10	Critical care	Degree
**P 15**	30-39	Female	11-20	Specialist role	Postgraduate certificate
**P 16**	30-39	Female	6-10	Critical care	Degree
**P 17**	40-49	Female	21-30	Critical care	Diploma
**P 18**	40-49	Female	11-20	Critical care	Diploma
**P 19**	20-29	Female	0-5	Critical care	Degree
**P 20**	20-29	Female	6-10	Critical care	Degree
**P 21**	20-29	Female	0-5	Critical care	Degree
**P 22**	30-39	Female	6-10	Critical care	Degree
**P 23**	40-49	Female	11-20	Specialist role	Degree
**P 24**	30-39	Male	6-10	Critical care	Degree
**P 25**	30-39	Male	0-5	Critical care	Degree
**P 26**	30-39	Female	6-10	Critical care	Degree

### Categories of description

After rigorous analysis, four categories of description were identified that represent qualitatively different ways of experiencing sepsis recognition: (A) recognise sepsis by following protocol-based care, (B) recognise sepsis by following disease-specific care, (C) recognise sepsis according to emergent care, and (D) recognise sepsis according to emergency care. This is consistent with the theoretical basis of phenomenography itself, which assumes that only a limited number of ways of understanding a phenomenon exist — commonly four or five variants [15].

The categories emerged from diverse clinical contexts, ranging from the simple (with well-known cause/effect links) to the highly complex (webs and loops of cause/effect with unpredictable, emergent properties). The complexity of these contexts shaped participants’ reasoning processes, thus indicating a strong relationship between the way of recognising sepsis and the context in which it occurs.

### Data display

As shown in Table 3, the raw phenomenographic content of each category is presented alongside the Cynefin-informed structure subsequently used to organise and present that content according to contextual complexity [17].

**Table 3:** Phenomenographic content and Cynefin-informed organisation of the categories of sepsis recognition.

Attribute	A: Protocol-based care	B: Disease-specific care	C: Emergent care	D: Emergency care
**Raw phenomenographic content**	Protocolised approach to disease; the human body viewed as an object compared against pre-agreed criteria (normal/abnormal observation ranges).	Reductionist approach to disease; the human body viewed as the sum of distinct parts, which can develop isolated diseases.	Shift in focus from disease to patient; recognition of non-linear, frequently unseen connections between variables, manifesting unpredictably.	Shift to an emergency approach; life-saving measures take precedence. The aim is to decrease exposure to the chaotic context and lower uncertainty.
**Availability of data**	Facts, rules and procedures	Facts, rules and procedures	Difficult to define, tackle and solve	Uncertain and constantly evolving
**Predictability**	Stable and predictable by everyone	Stable and predictable by experts	Unpredictable and uncertain	Unstable
**Cause-and-effect relationship**	Clear cause-and-effect relationship	Discernible with analysis	May be understood, but only in retrospect	Cannot be determined; a life-saving action comes first
**Reasoning strategy**	Rule-based reasoning	Analytical reasoning	Reasoning in terms of interconnected systems	Transient
**Pattern of actions**	Sense, categorise, and respond	Sense, analyse, and respond	Probe, sense, and respond	Act, sense, and respond
**Cynefin domain**	Simple	Complicated	Complex	Chaotic

The raw phenomenographic content presents the descriptive content of the four categories as established through the phenomenographic analysis before any mapping to the Cynefin framework was undertaken. The subsequent rows show the later conceptual interpretation and alignment of these categories with characteristics of the corresponding Cynefin domains: protocol-based care (Simple), disease-specific care (Complicated), emergent care (Complex) and emergency care (Chaotic).

The distinction between emergent care and emergency care lies in the nature of the situation: emergent care reflects complex situations requiring adaptive and exploratory responses, whereas emergency care reflects chaotic situations requiring immediate and decisive action.

As shown in Table 3, the CynefIn framework that emerged from complexity science [18], provided a structure that accommodated the varying levels of complexity observed in our data. The framework enabled systematic organisation of the categories and their corresponding reasoning pathways based on the complexity of clinical contexts, accounting for the nature of cause-and-effect relationships, the degree of predictability, and the availability of data in each context, as detailed in the section below.

### Theme A: Recognise sepsis by following protocol-based care

#### Context.

**Predictability, availability of data, and cause-and-effect relationship**. Participants ascribed protocol-based care to recognition of sepsis based on information that was known, explicit, easy to define, readily available, and matched the criteria in the protocol. Participants assigned probability to an outcome in advance and automatically initiated a definite course of action for the presented problem. Here, participants replicated a fixed protocolised approach with other patients, assuming the occurrence of repeating patterns and consistent events.

The following excerpts provide examples in which recognition of sepsis did not depend on noticing all factual information about the patient, but instead depended on the nurses’ ability to identify those that match the criteria in the protocol.

*“So, a little like the qSOFA … So, it was if you had any two out of the three, so I think resp. over 22, a systolic blood pressure below 100 or a new altered conscious state.” (participant 21)*

*“…like what I said when patients get there to use a....get set of observations because it’s good to sort of look at the numbers, make sure that that you sort of*

*identify any potential risk to the patient and get a baseline for their numbers and always using the NEWS 2 score two because it’s what we use in the Trust.” (participant 06)*

### Process

#### Reasoning strategy.

In this context, participants applied rule-based reasoning by identifying deviations of physiological values from expected norms, through matching them against the predetermined criteria of a protocol. Participants used predetermined criteria to guide the selection and implementation of actions.

As seen in the following excerpts, rule-based reasoning was also applied to protocol thresholds and the timescale within which interventions needed to be delivered. Nurses described predetermined rules and objectively defined timeframes for action, as indicated by the protocol, within which they were expected to respond.

*“… the rule is sort of an hour from door to needle, so we would hope that within that time of identifying somebody with sepsis, and especially if they’re neutropenic as well, and give them antibiotics in that time.” (participant 06)*

*“If the patient has two out of these three signs, like I say, resp, blood pressure and conscious state of the patient, the mental state of the patient, you know, if you ticked two of those boxes … it quite clearly would say, if this is the case go on to step two ….” (participant 21)*

#### Pattern of actions.

The appropriate pattern of action was sense, categorise, and respond. A certain level of comprehension was viewed as possible prior to action. Participants sensed that something was wrong with their patient, categorised findings according to protocol, and then grounded their responses in established best practice.

As illustrated in the excerpts below, participants described first suspecting sepsis, then matching findings to protocol criteria, and subsequently initiating treatment or further investigation.

*“If I am suspecting sepsis, I would always kind of follow the same, follow the same plan really.” (participant 11)*

*“So, a little like the qSOFA … I think if it was any two out of those three. If I remember rightly then you would treat or investigate whether they did have sepsis.” (participant 21)*

### Theme B: Recognise sepsis by following disease-specific care

#### Context.

**Cause-and-effect relationship, availability of data, and predictability**. Participants ascribed disease-specific care to recognition of sepsis based on a cause-and-effect relationship that was knowable. By considering multiple causal factors that aligned linearly and synergistically with symptoms, there was a possibility of forming a list of probable hypotheses compatible with the symptoms. Here, participants emphasised information that was definable, observable, and discoverable, even if not immediately obvious. However, the outcomes were predictable by experts and via the inclusion of a multidisciplinary team with the right expertise.

As illustrated in the excerpts below, participants described a knowable cause-and-effect relationship in which observable symptoms, such as a chesty presentation or urinary symptoms, could be linked to several plausible causes, allowing hypotheses to be formed and refined through assessment.

*“If somebody when I examined, if somebody is chesty, so obviously I would focus on their chest. I would, you know if they were complaining of urinary symptoms, I would obviously think, you know, my second observation would be, you know, they’ve got urinary symptoms.” (participant 12)*

*“If I suspect that someone has sepsis, I would go through my Sepsis Six… And then kind of like urine sample or chest x-ray depending on what I thought the source was.” (participant 11)*

### Process

#### Reasoning strategy.

In this context, a set of hypotheses was formed by assigning probabilities to the clinical problem, which are linear and synergistically compatible with symptoms (if A, then B, and thus next C). Hypotheses were ruled out or ruled in by breaking them down and analysing their components separately. The hypotheses were refined by the analytical approach.

As illustrated in the excerpts below, participants described ruling things out by analysing separate components of the clinical problem, such as urine or blood samples, and progressively ticking potential explanations off the list until the most probable hypothesis remained.

*“I think it was by ruling things out, because by the time you’ve started sending things that weren’t coming back with anything, like the urine was sent and that came back with nothing in it, you were kind of ticking off all of the list of things it could potentially be.” (participant 23)*

*“… if it is like if it is if you have a doubt about what your patient is experiencing, …. maybe more investigations could be done, like blood samples to be taken, … so get, you know, blood samples taken and analyse it at the earliest enough.” (participant 03)*

#### Pattern of actions.

The appropriate pattern of action was sense, analyse and respond. A certain level of comprehension of a clinical problem was still considered possible before action. However, as there were multiple causal factors for the clinical problem, action was not used to categorise the problem but rather became a means of analysis, performed by an expert with the right kind of expertise, who could interpret the outcomes and respond accordingly.

As illustrated in the excerpts below, this pattern of action involved nurses reaching a sufficient level of comprehension of the underlying causality, then turning to a doctor to investigate blood results, undertake further analysis, and plan the subsequent response.

*“… I think she’s septic. The doctor then needing to, …needed to be cannulated for fluids, bloods would need to be taken.” (participant 08)*

*“As long as you got an idea in your head of what it could be, then you know what to then go and do. I just think you should always consider every route and then if you go no that’s not that, then at least you’ve ticked it off the list of potentials and then you can plan from there.” (participant 08)*

### Theme C: Recognise sepsis by following emergent care

#### Context.

**Availability of data, cause-and-effect relationship, and predictability**. Participants ascribed emergent care to early recognition of sepsis based on information that was difficult to define or contradictory and subject to competing interpretations due to multiple causes contributing to observed outcomes.

Here, participants could not assign probability to an outcome in advance, nor could they automatically initiate a definite course of action for the presented problem. Such uncertainty meant that information could only be approximated and used to guide an experimental process.

As reflected in the excerpts below, nurses described multiple sources of complexity within clinical information, such as subtle changes that were difficult to define, overlapping signs and symptoms with alternative explanations, underlying drivers that were not immediately visible, and presentations that did not display classic signs or meet standardised sepsis criteria.

*“… it’s hard to explain in terms of just looking at the patient, …so for example, they look different ….” (participant 24)*

*“Because a lot of these signs and symptoms can be related to, you know say people have taken drugs in the community or they could have fallen and just banged their head. So, some of the things all overlap.” (participant 22)*

*“They look like there is something underlying what’s driving this. … might not be showing you the classic signs or might not be hitting in the numbers like sepsis six ….” (participant 24)*

*“… sometimes I think in the initial stages you’re trying to work out… If they’re like speaking then you can kind of ask them, … it’s like a bit of a guessing game to work out what you think it could be. Sometimes like medications can make people a bit confused. Sometimes it can cause them … that they don’t just react properly.” (participant 22)*

### Process

#### Reasoning strategy.

In this context, participants used reasoning in terms of interconnected systems, using incomplete observations to generate possible explanations early in the process. It relied on the ability to construct a diversified network of connections between clinical features. The connections were nonlinear, emerged from seemingly unrelated observations, and were not always visible. However, participants emphasised the need to remain open-minded as the connections constituted the essence of solvable problems.

As reflected in the excerpts below, participants described reasoning across interconnected clinical features rather than interpreting observations in isolation, such as considering “everything as a whole”, exploring “the causes”, and remaining open to “other reasons”. These other reasons included recent surgery, medication effects or other contextual factors that may point to nonlinear and seemingly unrelated causes of clinical deterioration.

*“… I look at, you know, everything as a whole. You know, I wouldn’t just like look at like you know a patient’s sleep pattern and just like, you know, not sleeping, just feeling tired. I look at the causes ….” (participant 12)*

*“… they’ve got a temperature and they have not passed urine, but there would be other reasons why that would happen, like I say, for example, the patient had been into theatre, so, they often don’t pass urine for a bit of time afterwards ….” (participant 16)*

#### Pattern of actions.

To ensure the plausible explanation was supported by data and validated against implementation outcomes, participants probed the explanations through observations, experiments or other evidence, sensed emerging patterns, and evaluated the impact and demand of their intervention. Here, key information was not observable but latent and emergent; therefore, action served as an experimental measure for targeting insight.

As reflected in the excerpts below, participants described a pattern of action in which interventions such as temperature management, pain relief, or fluid replacement were used as experimental measures to probe plausible explanations, while observing whether the patient’s response supported or challenged the plausible explanation.

*“… could be a possible infection, so it is just trying to give them… so paracetamol, try to put a fan on them, try and remove blankets, just to bring that temperature down naturally. For, you know, extreme pain, try to get the pain relief on board as quick as possible.” (participant 22)*

*“… if I felt they were fluid depleted, I would want to know if they had any fluid replacement, what their fluid status was. If they responded to that, if they had any tachycardia that didn’t respond to fluid.” (participant 09)*

Having evaluated possible explanations for the patient’s physiological presentation, participants adopted or adapted management strategies depending on the way a patient’s presentation progressed under their care.

*“Bring the heart rate down. Sometimes it could be a response to pain or temperature. It could be they might need an antihypertensive drug because their blood pressure has gone up as well.” (participant 22)*

As illustrated in the excerpt above, the participant evaluated provisional explanations by considering whether the raised heart rate was related to pain or temperature, while adapting interventions according to the patient’s physiological presentation.

Participants were constantly comparing provisional explanations with emerging information, including intervention responses, symptom progression, and laboratory tests. Each confirmatory or negative test result informed the evolving interpretation of the patient’s condition.

*“… it’s just constant assessment of whatever we’re instituting. Whether it be inotropic support, or fluids, or bringing a patient’s temperature down or constant just assessment of the patient, you know the lactate, blood gases. How the patient looks in themselves.” (participant 09)*

### Theme D: Recognise sepsis by following emergency care

#### Context.

**Cause-and-effect relationship, availability of data, and predictability**. Participants ascribed emergency care to recognition of sepsis based on cause-and-effect relationships that could not be immediately determined. The information emphasised in decision-making was constantly evolving, depending on the outcomes of resuscitation procedures. The context lacked predictability, partially due to the unpredictable outcomes of resuscitation procedures.

As reflected in the excerpts below, participants described a context in which they would not initially have suspected sepsis because cause-and-effect relationships were difficult to determine during immediate deterioration, such as low blood pressure, reduced heart function or the need for intubation. The context lacked predictability, as information changed rapidly in response to resuscitation, requiring clinicians to assess what was or was not working and decide “what do we try next?”

*“… if a patient experiences low heartbeat, low blood pressure, or like something which is actually keeping the right organ function, there is always a possibility that they can go into organ without having it sophisticated in time ….” (participant 03)*

*“There was immediate feedback as well from the doctor. Like, oh this is not working, that’s why it’s not working, what do we try next?” (participant 10)*

### Process

#### Pattern of actions.

In this context, the pattern of actions consisted of act, sense, and respond. Here, action was not an experimental measure for targeting insight, but instead was an emergency measure for stabilisation.

As demonstrated in the excerpts below, participants described situations in which immediate deterioration required urgent action before sepsis could be fully analysed or recognised. The priority was to stabilise the patient, prevent or reverse deterioration, and only then, where possible, identify the root cause of the problem.

*“… when you come when you receive a patient like you know, going or having very, needing intubation say for example, so you wouldn’t think that would be a cause for sepsis ….” (participant 03)*

*“There was no space for analysing the recognition of sepsis” (participant 10)*

*“If you stop deteriorating and you reverse the process, and if you ideally can identify the root cause of this, that’s the big help.” (participant 01)*

#### Reasoning strategy.

This line of reasoning was transient. Following a life-saving action, participants sensed the spot where stability was present or absent and responded by working towards changing the situation from emergency care to emergent care to understand the problem, or towards disease-specific care to address the problem.

Following a life-saving action, such as treating low blood pressure with fluids or inotropes, nurses described shifting from stabilising the patient towards understanding the wider problem and recognising patterns of whole-system deterioration. Evidence for these claims is demonstrated in the excerpts below.

*“… if we are able to identify these symptoms in time, and treat it, like, say for example, if we have a low blood pressure, treat it with fluid on this occasion and also you know hypertonic solution or with the medications to manage their blood pressure, you know, inotropes and identify it at the same time.” (participant 03)*

*“… when you receive a patient …, needing intubation say for example, so you wouldn’t think that would be a cause for sepsis but …for these patients who are going into sepsis you are going to note anything else apart from neuro problems, it is not a respiratory problem, it is like a whole of the systems maybe needing help with.” (participant 03)*

### What it takes to recognise sepsis

Four different ways of recognising sepsis were revealed. These categories were not mutually exclusive classifications of individual participants or whole transcripts. Individual accounts frequently contained more than one way of recognising sepsis, and participants could move between reasoning approaches as the clinical context and available information changed.

While some participants predominantly relied on one form of reasoning, others described sepsis recognition as a personalised process that drew on a series of different reasoning strategies. Participants also shifted between forms of reasoning depending on context. This reflected a context-driven and personalised approach to sepsis recognition. These different ways of recognising sepsis were distributed across different levels of years of practice.

The complete individual-level mapping is provided in S3 Table. The mapping demonstrates that more than one way of recognising sepsis could be present within an individual account. Protocol-based, disease-specific and emergent care appeared as either predominant or less dominant ways of recognising sepsis, whereas emergency care appeared only as a less dominant approach. These ways of recognising sepsis were evident across different levels of professional experience, with no evidence of a stepwise progression from less to more complex approaches with increasing years of practice.

Examples from participants’ accounts showed movement between different forms of reasoning, including from protocol-based to disease-specific reasoning and from emergency to emergent and disease-specific reasoning. Participant 21 illustrated movement from protocol-based to disease-specific reasoning, with protocol-based reasoning acting as a point of departure followed by further analytical investigation to assess whether the patient had sepsis.

*“So, a little like the qSOFA that they have at [name of hospital]. So, it was if you had any two out of the three, so I think resp. over 22, a systolic blood pressure below 100 or a new altered conscious state, state of consciousness, think if it was any two out of those three. If I remember rightly then you would treat or investigate whether they did have sepsis.” (participant 21)*

Participant 03’s account demonstrated that the ability to recognise sepsis could involve three distinct forms of reasoning. Initial emergency care acted as a point of departure, but recognition then involved incorporating other forms of reasoning to generate knowledge and recognise sepsis. The first form of reasoning was based on taking immediate action to stabilise the patient and observe patterns of deterioration (emergency care). This then moved towards forming connections among the various systems according to emergent patterns (emergent care), and was followed by calling in experts and analysing multiple options (disease-specific care).

*“... when you receive a patient like you know, going or having very, needing intubation say for example, so you wouldn’t think that would be a cause for sepsis but if you sometimes what I think like when you receive a patient, by looking at them, they are clammy, like a laboured breathing, less urine output, I have had these patients several times, for these patients who are going into sepsis you are going to note anything else apart from neuro problems, it is not a respiratory problem, it is like a whole of the systems maybe needing help with.” (participant 03)*

*“I mean, it’s a team work, so if it is like if it is if you have a doubt about what your patient is experiencing, it should be alerted to the higher level and maybe more investigations could be done, like blood samples to be taken, anyway this will be done if the patient is coming into this kind of situation anyway, so get, you know, blood samples taken and analyse it at the earliest enough, ....” (participant 03)*

Participant 03’s account highlights the difference between recognising sepsis retrospectively, when complete patient information may be available, and recognising sepsis prospectively through a developing clinical picture while data are still missing or evolving.

Recognition was not described as passive waiting for complete information to become available. Rather, it involved an active, multi-layered reasoning process in which nurses formed connections across physiological systems while data were still missing or evolving, using emergency, emergent and disease-specific reasoning processes to support timely recognition and intervention.

In contrast, while some nurses recognised that different contexts required distinct responses, in other accounts contextual uncertainty or complexity was present without a corresponding change in reasoning or clinical action. For example, Participant 11 described continuing to use a protocol-based approach despite uncertainty about the underlying cause of the patient’s presentation: “I would pretty much always follow kind of the guidelines in the Sepsis Six, even if kind of… I’m not entirely sure what is causing it.” Here, uncertainty regarding causation coexisted with protocol-based reasoning and action. When considered in relation to the Cynefin framework, this combination did not map onto any one of the four domains: Clear/Simple, Complicated, Complex, or Chaotic because the contextual characteristics did not correspond with the distinct response associated with that context. Such instances were retained as part of the phenomenographic variation and suggest that recognising contextual uncertainty or complexity did not necessarily translate into an ability to adapt reasoning strategies or clinical actions accordingly.

*“ I think really if I am suspecting, if I am suspecting sepsis, I would always kind of follow the same, follow the same plan really, so, I would pretty much always follow kind of the guidelines in the Sepsis Six, even if kind of… I’m not entirely sure what is causing it.” (participant 11)*

## Discussion

In this study, we derived the first complexity-informed framework for sepsis recognition to date to guide an individualised approach to sepsis recognition, using data from heterogeneous clinical contexts. For this, we undertook the effort to examine the heterogeneity of clinical contexts nurses encountered in clinical practice, especially complex contexts where the data are sparse [6,19]. Using these data, diverse groups of clinical contexts were identified, ranging from the simple (with well-known cause/effect links) to the highly complex (webs and loops of cause/effect with unpredictable, emergent properties). Alongside this, differences in how these contexts impacted specific practice were identified. When systematically organised, that is, classified into distinct categories based on the complexity of clinical contexts, we derived a multidimensional framework that can be rendered clinically useful. Rather than relying on an identical strategy for all patients, clinical decisions can be personalised, with the strategy selected based on the specific characteristics of the individual context, including cause-and-effect relationships, predictability, and availability of data.

Sepsis is one of the most heterogeneous and challenging conditions to detect in ICU and is the leading cause of mortality in critically ill patients [20]. Therefore, the possible benefits of a nuanced framework applicable to a broad spectrum of clinical contexts, and which can underpin and support a personalised approach to sepsis recognition, are manifold.

Multiple studies have attempted to address this problem using sepsis screening tools, but many of them are based on standardised criteria [7,8], limiting their applicability to complex patients who do not fit specific criteria but may still benefit from advances in sepsis treatment [6,10].

Consequently, the fixed approach to sepsis recognition using screening tools has been found to have inconsistent efficacy. One of the most widely implemented screening tools for sepsis recognition was recently found to perform poorly [7,8], reflecting its inability to capture the heterogeneity of clinical contexts encountered clinically in sepsis [21]. This emphasises the fundamental importance of our study.

In contrast, the present study examined the characteristics of different clinical contexts encountered in practice, especially complex contexts where the data are sparse [6]. This illustrated how these differences in context impacted the practice, rendering our approach more flexible when a certain way of recognising sepsis was not adequate to accomplish the required outcome. Utilising this approach, we described how the variability of clinical issues served to direct clinical management. This enabled nurses to decide whether the sepsis protocols applied to the individual patient at all and, if so, how they should be integrated into a clinical decision.

For example, when information was known, explicit, readily available and matched protocol criteria, protocol-based care directed nurses towards predetermined actions to recognise and respond to sepsis. When cause-and-effect relationships were knowable, disease-specific care directed nurses towards considering multiple causal factors, aligning them with symptoms, and forming probable hypotheses to guide further assessment and investigation. When information was difficult to define, contradictory or open to competing interpretations, emergent care directed nurses towards reasoning in terms of interconnected systems and probing uncertainty through observations, interventions or other evidence. When cause-and-effect relationships could not be immediately determined and the patient was unstable, emergency care directed nurses towards life-saving action to stabilise the situation before moving towards emergent care to understand the problem or disease-specific care to address it.

By proposing a framework grounded in these findings, our work aimed to complement protocol-based care while simultaneously promoting personalised care. This concept has been proposed in the literature, but has not had supporting evidence from research [6,19]. This fact, together with the personalised approach, underpins the potential value of the framework we propose.

### Implications for clinical practice and education

Our findings have implications for sepsis recognition in clinical practice, particularly as healthcare shifts toward individualised care [6]. The application of standardised sepsis criteria with established diagnostic thresholds across a broad spectrum of clinical situations, without consideration of heterogeneous populations of patients with sepsis presenting at different stages of clinical progression, may be of limited value. Sepsis criteria can remain an important point of departure, but should no longer be considered the only one. We propose optimising sepsis recognition in a more personalised manner through a framework that complements protocol-based care while simultaneously promoting individualised clinical reasoning. This framework shifts sepsis recognition from determining whether a patient has crossed a particular threshold to recognising clinical trajectory, including evolving deterioration and responses to intervention.

The framework may also inform the refinement of sepsis screening tools and escalation pathways. For example, screening tools could incorporate explicit prompts that encourage nurses to consider broader contextual cues when protocol-based criteria are insufficient and more emergent reasoning is required. In practice, the framework may support nurses in recognising when a shift between reasoning approaches is required over the course of one patient’s care. For instance, a patient may initially present with a clear disease pattern that aligns with protocol-based care, whereby nurses predominantly rely on standardised sepsis criteria. However, as time continues to pass in assessment without results, the framework may support nurses in moving towards broader and more emergent forms of reasoning to guide timely recognition of sepsis. Consequently, the framework may support nurses in thinking more comprehensively about sepsis recognition, using a combination of the four reasoning pathways. This approach demands educational support; however, unless we learn to adopt the approach that is appropriate to the level of clinical complexity, significant improvements in sepsis recognition might not be seen.

The four identified approaches, protocol-based, disease-specific, emergent and emergency care, may provide a useful framework for structuring training, simulation or reflective debriefings about sepsis recognition. The framework may inform simulation design by incorporating scenarios with varying levels of clinical ambiguity and complexity, exposing learners to presentations that require movement between different reasoning approaches according to clinical context. It may also support reflective debriefing by helping educators examine how nurses adapt their reasoning in response to contextual uncertainty and evolving patient presentation. This may assist the development of context-responsive reasoning beyond protocol-based assessment alone.

### Limitations

An important limitation of this study was that the sample composition was uneven, with the majority of nurses recruited from intensive care across two hospitals within one region of England. This ICU sample may have contributed to the prominence of emergent and emergency care themes, as intensive care nurses’ prolonged time at the bedside may have contributed to greater sensitivity to tacit and non-specific clinical cues. However, intensive care nurses also brought experience from a range of clinical settings, which may have broadened the range of clinical experiences represented in the data. Nevertheless, as the framework remains derived from this specific sample and clinical context, further research is required to establish its transferability to other settings, such as emergency departments, community settings and non-UK healthcare systems. The availability of data identified as an important characteristic of clinical context may also be influenced by the technical infrastructure of the clinical setting, rather than by nurses’ reasoning processes alone. Furthermore, movement between the identified reasoning approaches may be structurally constrained by the clinical context; ward nurses without access to continuous monitoring or rapid laboratory results may face greater barriers to engaging in disease-specific analytical reasoning than their ICU counterparts and may consequently rely more heavily on protocol-based or emergent reasoning under conditions of uncertainty. This may influence how the identified reasoning approaches are applied across different clinical settings and should be considered when interpreting the transferability of the proposed framework.

Furthermore, movement between Cynefin domains may be structurally constrained by the technical infrastructure of the deployment context; ward nurses without access to continuous monitoring or rapid laboratory results may face greater barriers to engaging in disease-specific or emergent reasoning than their ICU counterparts, which may limit the immediate applicability of the framework in lower-acuity settings.

It was not possible to determine whether there were associations between nurses’ training, experience, clinical context and the processes used to recognise sepsis. Phenomenography focuses on variation in experiences, rather than demographic subgroup comparisons. Although this study found that four different ways of recognising sepsis were distributed across nurses with different years of practice, the study was not designed to establish whether these categories were influenced by training, experience or clinical speciality. Even the emergent care category, which involves the recognition of evolving patterns, was not limited to nurses with longer experience. Further research using comparative designs is required to explore whether factors such as training, years of experience, or clinical speciality influence these categories of sepsis recognition.

It was not possible to determine whether broader contextual factors influenced sepsis recognition, as this study explored sepsis recognition in relation to immediate characteristics of clinical contexts, such as predictability of events, cause-and-effect relationships, and availability of data. Further research is needed to examine whether broader contextual factors, including patient population characteristics, pathogens present in the wider environment, and access to healthcare, influence sepsis recognition across different clinical contexts.

As the data were generated from nurses working in two hospitals within one region of England, the framework remains context-derived, and its transferability to other clinical, geographical and non-UK healthcare settings requires further testing.

In addition, this study was limited to examining how nurses recognised and reasoned about possible sepsis. It did not examine how frequently sepsis as a presumed diagnosis at the time of presentation was ultimately the correct and final diagnosis, confirmed at the end of an admission when other possibilities had been considered and ruled out. This warrants further investigation into the frequency with which nursing perceptions that a patient had sepsis, or did not have sepsis, correspond with the final diagnosis.

## Conclusions

Despite its apparent simplicity, sepsis recognition is complex and context-dependent. Rather than relying on an identical strategy for all patients, effective clinical decisions require personalisation based on the characteristics of the clinical contexts. Our findings show that considering the availability of data, predictability, and cause-and-effect relationships can inform the selection of an appropriate approach to sepsis recognition. Prospective research is required to validate our findings.

## Supporting information

S1 Checklist Standards for Reporting Qualitative Research (SRQR) checklist. (DOCX)

S2 File Interview Schedule. (DOCX)

S3 Table Nurses’ predominant (++) and less dominant (+) ways of recognising sepsis. (DOCX)
