# Supplementary material for: Understanding the processes involved in sepsis recognition across clinical contexts to guide individualised management: A phenomenographic study
Source: PLoS One. 2026 Oct 27;21(10):e0360913. doi: 10.1371/journal.pone.0360913 (PMC13712736; doi:10.1371/journal.pone.0360913)
Supplement: Supplementary file 2 [file pone.0360913.s002.docx]

**Interview Schedule**

• Introduction

• Welcome the participant

• Reinforce the purpose of the research study and remind the participant of the ethical rules and use of data.

• Obtain written consent to record and use the interview data

**Use the questions as a guide:**

• Can you tell me what your role is and little about your history as a nurse?

• Based on your experiences so far, what does learning how to recognise sepsis early mean to you?

• If the participant asks for further explanation:

What sort of things do you do? What are you trying to achieve?

• Can you give me a concrete example of experience?

• How do you judge that something that you have done to early recognise sepsis has been successful?

• Can you give me a concrete example of something you have done to help you develop or grow your knowledge and skills?

These questions are a guide and will be used at each interview. Depending on the responses given, they may be expanded upon using the following probing questions.

• How did you go about that?

• Why did you do it that way?

• What did you gain or hope to gain from it?

• Why do you do that?

• What where you hoping to achieve?

Thank the participant for their contribution.
