# Supplementary material for: Understanding the processes involved in sepsis recognition across clinical contexts to guide individualised management: A phenomenographic study
Source: PLoS One. 2026 Oct 27;21(10):e0360913. doi: 10.1371/journal.pone.0360913 (PMC13712736; doi:10.1371/journal.pone.0360913)
Supplement: Supplementary file 3 [file pone.0360913.s003.docx]

**S3 Table. Twenty-six qualified nurses’ predominant (++) and less dominant (+) ways of recognising sepsis across four categories.**

| Participant | Gender | Years of practice | Protocol-based care | Disease-specific care | Emergent care | Emergency care |
| --- | --- | --- | --- | --- | --- | --- |
| P 06 | Female | <5 | ++ | + |  |  |
| P 21 | Female | <5 | ++ | + |  |  |
| P 08 | Female | <5 | + | ++ |  |  |
| P 05 | Female | <5 | + | ++ | + |  |
| P 07 | Female | <5 | + | + | ++ |  |
| P 04 | Female | <5 | + | ++ | + |  |
| P 19 | Female | <5 | + | ++ |  |  |
| P 25 | Male | <5 | + | ++ |  |  |
| P 24 | Male | 5–10 | + | + | ++ |  |
| P 02 | Female | 5–10 | + | ++ | + |  |
| P 10 | Female | 5–10 |  |  | ++ | + |
| P 13 | Female | 5–10 | + | ++ |  |  |
| P 14 | Female | 5–10 | + | ++ |  |  |
| P 11 | Female | 5–10 | + | ++ | + |  |
| P 16 | Female | 5–10 | + | + | ++ |  |
| P 20 | Female | 5–10 | + | ++ |  |  |
| P 22 | Female | 5–10 |  | + | ++ |  |
| P 26 | Female | 5–10 | + | ++ |  |  |
| P 01 | Female | 15–20 | + | + | ++ |  |
| P 15 | Female | 15–20 | + | ++ | + |  |
| P 18 | Female | 15–20 |  | + | ++ | + |
| P 23 | Female | 15–20 | + | ++ |  |  |
| P 03 | Female | >20 | + | + | ++ | + |
| P 17 | Female | >20 |  | ++ | + |  |
| P 09 | Female | >20 | + | + | ++ | + |
| P 12 | Female | >20 | + | + | ++ |  |

S3 Table note: The symbol “++” indicates the participant’s predominant way of recognising sepsis. The symbol “+” indicates less dominant ways of recognising sepsis. Blank cells indicate that the category was not clearly evident in the participant’s account.
